# Association between patient-reported functional measures and incident falls

**DOI:** 10.1038/s41598-021-84557-3

**Published:** 2021-03-04

**Authors:** Wanfen Yip, Lixia Ge, Bee Hoon Heng, Woan Shin Tan

**Affiliations:** 1 Health Services and Outcomes Research, National Healthcare Group, 3 Fusionopolis Link #03-08, Nexus@one-north, Singapore, 138543 Singapore; 2 Geriatric Education and Research Institute, Singapore, Singapore

**Keywords:** Geriatrics, Health care

## Abstract

Lower extremity muscle strength, and functional limitations are important modifiable predictors of falls, but are often examined using performance based measures. We examined the association between self-reported physical function limitations, determined using Late-Life Function and Disability Instrument(LLFDI) and incident falls in community-dwelling elderly individuals. 283 older adults participants were included in this analysis. Physical function limitations were defined as a person’s difficulty in completing items of the lower extremity function domain and composite scores of the LLFDI. Information on falls was obtained through a standardised questionnaire. At one-year follow-up, 15.2% (43) of the participants experienced their first fall. In the multivariable analysis, individuals who reported difficulties in items of lower extremity function domain were more likely to experience a fall (incidence rate ratio[IRR]: ranging between 2.43 and 7.01; all P ≤ 0.046). In addition, decreasing advanced lower extremity function scores (IRR: 1.70, 95% confidence interval[CI]): 1.04, 2.78) and overall function component score (IRR: 2.05, 95% CI: 1.22, 3.44) were associated with higher risk of incident falls. Physical function limitations, determined using LLFDI, were associated with incident falls. Our findings provide further evidence that the LLFDI function component has the potential to be used as a self-assessment tool for fall risk.

## Introduction

Falls are among the most common and serious problems facing older adults^1^. According to the World Health Organization, globally, 28–35% of the older adults (≥ 65 years) sustain falls annually^2^. Falls are the main cause of injury, injury related disability, and death in older people, with 40–60% of falls resulting in fractures, or traumatic brain injuries^3–5^. Additionally, studies have reported that older adult fallers suffered functional decline and increased risk of subsequent falls^6–10^. The financial cost from fall-related injuries is also substantial. For example, in the United States, the estimated direct medical cost of fall related injuries among older people in 2012 was $30bn^11^, of which, 66% was attributable to injuries requiring hospital admission^12^. In view of the implications of falls in the older adult population, it is crucial for the early detection and interventions of older individuals at risk of falls.

While there are many risk factors for falls in older people, decreased balance control, weak lower extremity muscle power, and overall functional limitation have emerged as highly modifiable factors^2,13–15^. With increasing age, gait coordination, balance control and muscle strength and tone have been known to decrease^16^. This decrease in balance control and muscle strength limits overall physical function, and impairs an older adult’s ability to maintain balance after a slip, leading to higher likelihood of falls^16,17^. Multiple studies and randomised controlled trials have demonstrated that with appropriate exercise interventions, muscle strength^18–21^, overall physical function^22^, and balance^23,24^ can be improved in the older adults. As such, early detection of these modifiable risk factors is key in falls prevention.

Patient-reported or interviewer administered measures have been shown to be a reliable and accurate methodology for obtaining information on lower extremity function, balance confidence and overall functional status^25^. While there are a wide variety of tools available to measure an individual’s functional ability, a lack of sensitivity to change, and inability to capture full spectrum of functioning have limited their practical application in the community^26^. The Late-Life Function and Disability Instrument (LLFDI) is a validated tool developed to address the aforementioned limitations^25,27,28^ and to assess both functional limitations and disability of an individual in the community. Importantly, LLFDI correlates well with Short Physical Performance Battery, Senior Fitness Test (SFT) battery, and mobility tests^29^. LLFDI also has the benefit of lower cost and convenience, and is a useful substitute for physical performance tests when a self-report or interviewer administered measure is preferred^28^. Despite the advantages of the function component of the LLFDI, the association between the individual items of lower extremity function domain and incident falls as well as the predictive ability of the function component for incident falls have not been extensively investigated^29^.

Hence, in this study, we examined the associations of items of lower extremity function domain and composite scores of LLFDI with incident falls in a group of community**-**dwelling older adults. Findings from this study will provide further evidence that the function component of the LLFDI reflects an individual’s physical function limitations, thus can be used as a self-assessment tool for fall risk.

## Results

Table 1 compares the baseline characteristics between those who did not experience a fall and those who fell. In this sample of community**-**dwelling older adults, 43 individuals (15.2%) experienced their first fall during the year. Participants who fell at 1-year follow-up were older and more likely to have risk of malnutrition or presence of malnutrition at baseline. Those who fell were also more likely to have poorer baseline basic and advanced lower extremity function and poorer overall physical function at baseline.

**Table 1:** Baseline characteristics comparing those who did not fall to those who fell.

	Did not fall (n = 240) Mean (s.d.)/n (%)	Fell (n = 43) Mean (s.d.)/n (%)	P-value*
Age, years	72.1 (6.0)	75.5 (8.1)	**0.001**
Gender, male	121 (50.4)	19 (44.2)	0.452
Ethnic group, Chinese	208 (86.7)	37 (86.0)	0.913
**Living arrangement**			
Alone	42 (17.5)	7 (16.3)	0.087
With spouse	72 (30.0)	9 (20.9)	
With children or grandchildren	74 (30.8)	10 (23.3)	
With other relatives/friends/ other unrelated individuals	52 (21.7)	17 (39.5)	
Hypertension status, yes	144 (60.0)	26 (60.5)	0.958
Osteoarthritis, yes	46 (19.2)	12 (27.9)	0.338
Osteoporosis, yes	28 (11.7)	8 (18.6)	0.209
Stroke, yes	11 (4.6)	4 (9.3)	0.371
Dementia, yes	157 (65.4)	33 (76.7)	0.145
Depression, yes	3 (1.3)	1 (2.3)	0.582
Polypharmacy, yes	59 (24.8)	13 (31.0)	0.400
Presence of vision/hearing impairment, yes	23 (9.6)	7 (16.3)	0.189
Risk of malnutrition or presence of malnutrition, yes	21 (8.8)	9 (20.9)	**0.017**
Basic lower extremity functioning domain score	86.6 (17.0)	74.4 (22.4)	** < 0.001**
Advanced lower extremity functioning domain score	67.0 (23.0)	51.2 (23.7)	** < 0.001**
Overall function component score	74.3 (16.5)	62.0 (16.2)	** < 0.001**

*P-value for differences between those who did not fall and those who fell, by t-test or chi-square test as appropriate.

Bold values are statistically significant for P-value ≤ 0.05.

Table 2 shows the adjusted results for items in basic and advanced lower extremity functioning domain that were significantly associated with incident falls. Individuals who reported some difficulty or quite a lot of difficulty in performing the basic and advanced items were more likely to experience a fall (basic lower extremity IRR ranging between 3.13 and 7.01, all P ≤ 0.041; advanced lower extremity IRR ranging between 2.43 and 3.21, all P ≤ 0.046). Individuals who reported only little difficulty in items such as going up and down a flight of stairs using handrail, running 800 m or running a short distance to catch a bus were instead, more at risk of falls (IRR ranging between 2.65 and 5.14; all P ≤ 0.023). Items in the basic lower extremity function domain and advance lower extremity function domain that were not significantly associated with incident falls are presented in Supplementary table S2.

**Table 2:** Relationship between baseline individual items of lower extremity function (basic and advanced) and incident falls.

	IRR (95% CI)*	P-value
**Basic lower extremity functioning domain**		
*Opening a heavy, outside door*		
No difficulty	Reference	
A little difficulty	1.24 (0.48, 3.18)	0.660
Some difficulty	**3.13 (1.16, 8.45)**	**0.024**
Quite a lot of difficulty/cannot perform	1.29 (0.42, 4.00)	0.654
*Stepping up and down from a curb*		
No difficulty	Reference	
A little difficulty	1.09 (0.26, 4.57)	0.902
Some difficulty	1.82 (0.61 – 5.42)	0.280
Quite a lot of difficulty/cannot perform	**3.27 (1.05 – 10.16)**	**0.041**
*Using a step stool to reach into a high cabinet*		
No difficulty	Reference	
A little difficulty	1.33 (0.53, 3.33)	0.146
Some difficulty	**3.96 (1.53, 10.25)**	**0.005**
Quite a lot of difficulty/cannot perform	2.15 (0.77, 6.05)	0.146
*Bending over from a standing position to pick up a piece of clothing from the floor*		
No difficulty	Reference	
A little difficulty	1.04 (0.36, 4.84)	0.460
Some difficulty	**4.24 (1.40, 12.80)**	**0.010**
Quite a lot of difficulty/cannot perform	1.54 (0.49, 4.84)	0.460
*Reaching overhead while standing as if to pull a light cord*		
No difficulty	Reference	
A little difficulty	1.79 (0.72, 4.47)	0.212
Some difficulty	**7.01 (1.85, 26.48)**	**0.004**
Quite a lot of difficulty/cannot perform	1.25 (0.45, 3.50)	0.674
*Going up down a flight of stairs using handrail*		
No difficulty	Reference	
A little difficulty	**2.65 (1.30, 5.40)**	**0.008**
Some difficulty	1.14 (0.34, 3.82)	0.828
Quite a lot of difficulty/cannot perform	2.01 (0.73, 5.40)	0.177
**Advanced lower extremity functioning domain**		
*Going up and down a flight of stairs without using a handrail*		
No difficulty	Reference	
A little difficulty	1.31 (0.48, 3.61)	0.599
Some difficulty	1.90 (0.62, 5.87)	0.262
Quite a lot of difficulty/cannot perform	**2.43 (1.07, 5.52)**	**0.034**
*Walk on a slippery surface outdoors*		
No difficulty	Reference	
A little difficulty	0.82 (0.34, 1.95)	0.650
Some difficulty	1.42 (0.61, 3.26)	0.414
Quite a lot of difficulty/cannot perform	**2.52 (1.02, 6.22)**	**0.046**
*Getting up from the floor (as you were laying on the ground)*		
No difficulty	Reference	
A little difficulty	2.17 (0.82, 5.78)	0.120
Some difficulty	1.18 (0.43, 3.19)	0.747
Quite a lot of difficulty/cannot perform	**2.72 (1.06, 7.00)**	**0.038**
*Walking 1.6 km, taking rests as necessary*		
No difficulty	Reference	
A little difficulty	0.50 (0.12, 2.05)	0.333
Some difficulty	**3.21 (1.61, 6.41)**	**0.001**
Quite a lot of difficulty/cannot perform	1.91 (0.71, 5.14)	0.202
*Running a short distant to catch a bus*		
No difficulty	Reference	
A little difficulty	**2.91 (1.32, 6.44)**	**0.008**
Some difficulty	–	
Quite a lot of difficulty/cannot perform	1.78 (0.84, 3.74)	0.130
*Running 800 m or more*		
No difficulty	Reference	
A little difficulty	**5.14 (1.26, 21.03)**	**0.023**
Some difficulty	1.01 (0.17, 5.95)	0.998
Quite a lot of difficulty/cannot perform	2.64 (0.71, 9.86)	0.149

*IRR* incidence rate ratio, *CI* confidence interval.

*Adjusted for age, gender, ethnic group, living arrangement, hypertension, polypharmacy, nutritional status, self-reported depression, stroke, osteoarthritis, osteoporosis, vision/hearing impairment, and dementia.

Bold values are statistically significant for P-value ≤ 0.05.

Table 3 shows the association between baseline basic and advanced lower extremity scores and the overall function component score with the risk of incident falls. In the multivariable analysis, after adjusting for age, gender and ethnic group, lower scores in basic lower extremity functioning domain (IRR: 1.46, 95% CI: 1.13, 1.87), advanced lower extremity functioning domain (IRR: 1.58, 95% CI: 1.20, 2.06) and overall physical function (IRR: 1.79, 95% CI: 1.38, 2.33) were found to be positively associated with incident falls. The association between baseline advanced lower extremity functioning domain score and baseline overall function component score with incident falls persisted after further adjustment for living arrangement, hypertension, polypharmacy, nutritional status, self-reported history of depression, stroke, osteoarthritis, osteoporosis, vision/hearing impairment, and dementia. Specifically, with every standard deviation decrease (23.5 score unit) in advanced lower extremity functioning domain score, there was a 1.7 times increased risk of incident falls (95% CI: 1.04, 2.78). Similarly, with every standard deviation decrease (17.0 score unit) in overall function component score, there was a 2.05 times increased risk of incident falls (95% CI: 1.22, 3.44). However, the association between baseline lower extremity functioning domain score with incident falls became insignificant.

**Table 3:** Relationships between baseline lower extremity (basic and advanced) and overall function score with incident falls.

	IRR (95% CI)	P-value
**Model 1**		
Basic lower extremity functioning domain score, per 18.4 SD decrease	**1.46 (1.13, 1.87)**	**0.004**
Advanced lower extremity functioning domain score, per 23.5 SD decrease	**1.58 (1.20, 2.06)**	**0.001**
Overall function component score, per 17.0 SD decrease	**1.79 (1.38, 2.33)**	** < 0.001**
**Model 2**		
Basic lower extremity functioning domain score, per 18.4 SD decrease	1.51 (0.94, 2.42)	0.086
Advanced lower extremity functioning domain score, per 23.5 SD decrease	**1.70 (1.04, 2.78)**	**0.033**
Overall function component score per 17.0 SD decrease	**2.05 (1.22, 3.44)**	**0.006**

Overall function component score: made up of three sub domains, upper extremity basic lower extremity and advanced lower extremity; Model 1: adjusted for age, gender, ethnic group; Model 2: additionally, adjusted for living arrangement, hypertension, polypharmacy, nutritional status, self-reported history of depression, stroke, osteoarthritis, osteoporosis, vision/hearing impairment, and dementia.

*IRR* incidence rate ratio, *CI* confidence interval, *SD* standard deviation.

Bold values are statistically significant for P-value ≤ 0.05.

## Discussion

In this study of community**-**dwelling older adults, we observed significant associations between items in lower extremity functioning domain with incident falls. Importantly, we demonstrated that decreased advanced lower extremity function score and overall physical function score, were independently associated with risk of incident falls within a year. Together, these findings suggest that physical function limitations, measured using LLFDI are good predictors of falls and thus may potentially be used as a fall risk self-assessment tool for community**-**dwelling older adults.

Decreased muscle power and balance control is recognised as one of the key modifiable risk factors of falls. It is widely known that a decrease in lower muscle power and decreased balance control result in mobility and function limitations thereby increasing an individual’s risk of falls^16^. Thus, an important finding in our study is the association between items in lower extremity functioning domain (basic and advanced) with incident falls. We observed that the likelihood of falls was higher in the group of individuals who reported significant difficulty in activities (requiring balance control and adequate muscle power) such as stepping up and down a curb, basic walking, walking over a slippery surface when compared to those who had no difficulties. Studies that measured balance control and lower extremity muscle power using performance measures (e.g.: timed up and go test, gait speed, etc.) also reported similar findings. For example, in a randomised controlled trial that included 259 British community**-**dwelling older people, the authors reported that timed up and go test, a measure of lower muscle power and balance, was independently associated with future falls^30^. We further demonstrated the association of advanced lower extremity function domain score and overall physical function component score, which are composite scores of the individual items, with incident falls. In fact, our findings echo the results from the Boston RISE cohort study that involved 430 older adults, where authors reported lower overall function component score was predictive of incident falls over a period of 2 years (OR: 1.27, 95%CI: 1.04–1.54, P-value: 0.0162)^29^. Taken together, it is plausible that poorer advanced lower extremity function and overall physical function, measured using function component in LLFDI, is indicative of decline in lower extremity muscle function and functional limitation^31–33^ and therefore at higher risk of falling.

In addition to being a self-reported screening tool, practitioners can also use the function component to individualise fall preventive strategies based on an individual’s self-reported ability to perform an array of daily living tasks. In our study, we observed that individuals who reported little difficulty in doing physical activities such as running after a bus or 800 m and going up and down a flight of stairs using handrail were more likely to fall as compared to those who had no difficulty. Interestingly, no significant association between those who reported significant difficulty in doing these physical activities (running after a bus or 800 m and going up and down a flight of stairs using handrail) with incident falls was observed. A possibility could be that individuals who had difficulties in such physical activities will not attempt it, thus a lower likelihood of falls^34,35^. As such, for persons who do engage in physical activities such as hiking, running, emphasis on safe performance will help them to engage in these activities without harm, thus remaining active and independent. On the other hand, for individuals who report significant difficulties in performing tasks such as climbing stairs, stepping up curbs or basic walking, targeted interventions could be introduced to improve muscle power and balance control^19,36,37^.

The strengths of this study include the use of standardised questionnaire and validated tools to collect data on falls risk factors and covariates, thus allowing us to conduct a comprehensive evaluation of risk factors associated with incident falls. However, this study also has a few limitations. First, as fall information was acquired via questionnaire as self-reported falls, there might be recall bias which may result in under or over-reporting of falls. Second, we only collected data on falls but did not further collect information on the aetiologies of falls and fall-related consequences. Third, our multivariable model may be viewed as an over adjustment strategy due to the number of covariates included; the true IRR may likely be slightly higher that the values presented in the final model should our sample size be larger.

In summary, in this population of community**-**dwelling older adults, we demonstrated significant associations between items in lower extremity functioning domain (basic and advanced) as well as low scores in function component were predictive of incident falls. Our findings provide evidence that LLFDI function component has the potential to be used as a self-assessment tool for falls risk detection.

## Methods

The Population Health Index (PHI) Survey is a longitudinal survey to examine the health of community**-**dwelling adult population in the Central region of Singapore. Details of the study have been described previously^38^. In summary, the PHI survey used a standardised study protocol to collect information on demographic and socioeconomic characteristics, physical and mental health status, functional and nutritional status, cognition and medication usage^38^. A sampling frame of residential dwelling units was constructed by matching postal codes in the National Database on Dwellings in Singapore with the list of postal codes for the Central region^38^. Within each planning area, a sample of dwelling units was selected proportionately from defined dwelling type groups. One eligible household member (Singaporean or permanent residents aged 21 years and above, staying in the household for > 6 months) was randomly selected using the Kish grid^39,40^.

Between November 2015 and November 2016, trained interviewers carried out baseline face-to-face survey^38^. A total of 1,942 individuals participated in the baseline survey, of which, 1,526 participants completed one-year follow-up survey between November 2016 to December 2017 (response rate: 78.6%). For this study, we included only participants aged 65 years and above (N = 518). We then excluded participants with missing baseline information on living arrangement, dementia, medication, Mini Nutritional Assessment (MNA) score, self-reported history of falls, hypertension, osteoarthritis, osteoporosis, stroke, depression, vision and hearing impairment and incident falls. Participants who had experienced a fall prior to the baseline survey were also excluded. The final analytical sample comprised of 283 participants. Baseline characteristics of those who were included and excluded from the data analysis are shown in Supplementary table S1.

The study was approved by the research ethics committee of the National Healthcare Group. Written informed consent was obtained from each participant before enrolment and the conduct of the study adhered to the Declaration of Helsinki.

### Independent variable—late life function and disability instrument

Premised upon Nagi’s disablement framework^27^, the LLFDI was developed to be a comprehensive assessment of function and disability for older adults^26,41^. Items in LLFDI measure functional limitations and disability^26,41^. The function component evaluates self-reported difficulty in performing physical activities with the overall score reflecting an individual’s physical function. This function component is made up of three sub-domains measuring limitations in the upper extremity, basic lower extremity, and advanced lower extremity^26,27,41^. Advanced lower extremity function reflected activities that involve a high level of physical ability and endurance. On the other hand, basic lower extremity functioning composed of activities that primarily involved standing, stooping and fundamental walking activities^27^. Previous studies have demonstrated that the test–retest reliability of LLFDI Function summary subscale scores was extremely high (intraclass correlation coefficients: 0.91–0.98)^26^. Function summary scores were also reported to be highly correlated with Medical Outcomes Short Form-36 scores (Spearman’s rank correlation 0.74–0.86)^42^.

Items of the lower extremity function domain were used as independent variables in this study. Items were phrased, “How much difficulty do you have doing a particular activity without the help of someone else and without the use of assistive devices?” with response options ranging from “none” to “cannot do”^28^. Responses from the items were analysed as categorical variables. The three composite scores used as independent variables in this study were basic lower extremity score, advanced lower extremity score and the overall function component score. The scores were calibrated on a 0 to 100 scale and analysed as continuous variables, with higher scores indicating higher levels of functioning^28^. However, a 1-point change in composite score represents a very small change in physical function limitation of the lower extremity (basic and advanced) and overall function. Hence, standard deviation for each composite score was computed to reflect a more substantial change in physical function.

### Outcome variable—incident falls at 1-year

Information on falls was collected through a questionnaire that asked—“Did you have a fall in the past one year?” An incident fall was defined as the experience of a fall between the baseline and one-year follow-up survey but having no falls prior to baseline. A dichotomised variable (Yes—Fell; No—Did not fall) was used in the analyses.

### Covariates

Covariates included participants’ socio-demographic characteristics (age, gender, ethnic group, living arrangement), cognitive and sensory function, and self-reported physician-diagnosed chronic conditions. We specifically included medical conditions, sensory losses^2,13,15,43^, and malnutrition^15^ as these risk factors can result in mobility impairment, gait and balance deficit (e.g. vertigo/giddiness), which will predispose older adults to falls. Polypharmacy was also included as it has been reported to be associated with increased risk of falls^13^.

Medical conditions (hypertension, osteoarthritis, osteoporosis, stroke, dementia, depression); hearing or vision loss, and malnutrition were included as dichotomised variables (Yes/No). Nutritional status was assessed using the MNA^44,45^. A score of < 17 indicated malnutrition, and a score of 17–23.5 indicated a risk for malnutrition^46^. Polypharmacy was defined as an intake of three or more types of medications^28,47^. The presence of cognitive impairment was defined based on self-reported physician-diagnosed dementia or the Montreal cognitive assessment (MoCA), using education level-specific cut-off scores^48^. Vision/hearing impairment was defined as self-reported difficulties with vision (even with glasses) or difficulties with hearing or the use of a hearing aid.

### Statistical analysis

We compared baseline characteristics of those who did not fall and those who fell using independent sample t-test or Chi-square test for continuous and categorical variables respectively. Multivariable robust (modified) Poisson regression analyses were performed to examine the association between items of lower extremity function, domain composite scores (basic lower extremity score, advanced lower extremity score and overall function component score) and incident falls. In model 1, age, gender and ethnic group were adjusted. Adjustment of demographic factors (age, gender, ethnic group) allowed the examination of the association between physical function with incident falls while holding the demographic factors constant. In model 2, living arrangement, hypertension, polypharmacy, nutritional status, self-reported history of depression, stroke, osteoarthritis, osteoporosis, vision/hearing impairment, and dementia were additionally adjusted. Comorbid conditions and living arrangement have been reported to be associated with incident falls. Hence, in model 2, further adjustment of these confounding factors allowed us to examine the independent effect of physical function limitations on incident falls.

Adjusted incidence rate ratios (IRR) were estimated from multivariable models and reported with corresponding 95% confidence intervals (CIs).

All statistical analyses were performed using Stata 13.0 (StataCorp LP, College Station, TX).

## Supplementary Information

Supplementary Table S1.

Supplementary Table S2.
