# Supplementary material for: Association between patient-reported functional measures and incident falls
Source: Sci Rep. 2021 Mar 4;11:5201. doi: 10.1038/s41598-021-84557-3 (PMC7933133; doi:10.1038/s41598-021-84557-3)
Supplement: Supplementary file 1 — Supplementary Table S1. [file 41598_2021_84557_MOESM1_ESM.docx]

**Association between Patient-reported Functional Measures and Incident Falls**

Wanfen Yip^1^, PhD, Lixia Ge^1^, MSc, Bee Hoon Heng^1^,MSc, FAMS, Woan Shin Tan^1,2^, PhD

1. Health Services & Outcomes Research, National Healthcare Group, Singapore

2. Geriatric Education and Research Institute, Singapore

### Correspondence to: Dr Tan Woan Shin, Health Services & Outcomes Research, National Healthcare Group. 3 Fusionopolis Link #03-08, Nexus@one-north Singapore 138543 Tel: (65) 64966945 Fax: 65 (64966870). Email: Woan_Shin_Tan@nhg.com.sg

Manuscript Word Count: 2615 words

Supplementary

| **Supplementary table S1: Baseline characteristics comparing participants excluded and included in the study** | | | |
| --- | --- | --- | --- |
|  | **Excluded (n = 235)** | **Included (n = 283)** | **P-value*** |
|  | Mean (s.d.)/ n (%) | Mean (s.d.)/ n (%) |  |
| Age, years | 75.6 (7.8) | 72.6 (6.4) | **<0.001** |
| Gender, male | 92 (39.2) | 140 (49.5) | **0.019** |
| Ethnic group, Chinese | 1949 (82.6) | 245 (86.6) | **0.205** |
| Living arrangement |  |  |  |
| Alone | 50 (21.3) | 49 (17.3) | **0.017** |
| With spouse | 56 (23.8) | 81 (28.6) |  |
| With children or grandchildren | 49 (20.9) | 84 (29.7) |  |
| With other relatives/ friends/ other unrelated individuals | 80 (34.0) | 69 (24.4) |  |
| Hypertension status, yes | 148 (63.0) | 170 (60.1) | 0.416 |
| Osteoarthritis | 52 (22.1) | 58 (20.5) | 0.082 |
| Osteoporosis | 33 (14.0) | 36 (12.7) | **0.003** |
| Stroke, yes | 22 (9.4) | 15 (5.3) | **0.136** |
| Dementia, yes | 156 (83.0) | 190 (67.1) | **<0.001** |
| Depression, yes | 8 (3.4) | 4 (1.4) | 0.051 |
| Polypharmacy, yes | 64 (27.4) | 72 (25.7) | <0.675 |
| Vision/hearing impairment, yes | 34 (14.5) | 30 (10.6) | 0.183 |
| Risk of malnutrition or presence of malnutrition, yes | 18 (7.7) | 30 (10.6) | 0.250 |
| Previous history of falls, yes | 50 (21.3) | 0 | **<0.001** |
| Subsequent falls at follow-up, yes | 28 (18.8) | 43 (15.2) | 0.338 |
| Basic lower extremity functioning domain score | 75.0 (24.9) | 84.8 (18.4) | **<0.001** |
| Advanced lower extremity functioning domain score | 50.3 (27.5) | 64.6 (23.8) | **<0.001** |
| Overall function component score | 62.8 (18.5) | 72.4 (17.0) | **<0.001** |
| *P-value for differences between those who were included to those who were excluded, by t-test or chi-square test as appropriate | | | |
