# Supplementary material for: Association between patient-reported functional measures and incident falls
Source: Sci Rep. 2021 Mar 4;11:5201. doi: 10.1038/s41598-021-84557-3 (PMC7933133; doi:10.1038/s41598-021-84557-3)
Supplement: Supplementary file 2 — Supplementary Table S2. [file 41598_2021_84557_MOESM2_ESM.docx]

**Association between Patient-reported Functional Measures and Incident Falls**

Wanfen Yip^1^, PhD, Lixia Ge^1^, MSc, Bee Hoon Heng^1^,MSc, FAMS, Woan Shin Tan^1,2^, PhD

1. Health Services & Outcomes Research, National Healthcare Group, Singapore

2. Geriatric Education and Research Institute, Singapore

### Correspondence to: Dr Tan Woan Shin, Health Services & Outcomes Research, National Healthcare Group. 3 Fusionopolis Link #03-08, Nexus@one-north Singapore 138543 Tel: (65) 64966945 Fax: 65 (64966870). Email: Woan_Shin_Tan@nhg.com.sg

Manuscript Word Count: 2615 words

Supplementary

| **Supplementary table S2: Relationship between baseline individual items of lower extremity function (basic and advanced) and incident falls** | | |
| --- | --- | --- |
|  | **IRR (95% CI)*** | **P-value** |
| **Basic lower extremity functioning domain** |  |  |
|  |  |  |
| Taking a 1.6km brisk walk without stopping to rest |  |  |
| No difficulty | Reference |  |
| A little difficulty | 0.83 (0.27, 2.49) | 0.733 |
| Some difficulty | 1.22(0.49, 3.02) | 0.665 |
| Quite a lot of difficulty/ Cannot perform | 2.15 (0.88, 5.26) | 0.095 |
|  |  |  |
| Sitting down and standing up from a low soft couch |  |  |
| No difficulty | Reference |  |
| A little difficulty | 2.33 (0.90 – 6.03) | 0.081 |
| Some difficulty | 1.30 (0.46 – 3.65) | 0.619 |
| Quite a lot of difficulty/ Cannot perform | 0.56 (0.09 - 3.40) | 0.531 |
|  |  |  |
| Putting on and taking off a coat or jacket |  |  |
| No difficulty | Reference |  |
| A little difficulty | 0.97 (0.38 – 2.46) | 0.947 |
| Some difficulty | 1.81 (0.10 – 31.24) | 0.684 |
| Quite a lot of difficulty/ Cannot perform | 2.31 (0.46 – 10.65) | 0. 310 |
|  |  |  |
| Getting into and out of a car/taxi |  |  |
| No difficulty | Reference |  |
| A little difficulty | 1.16 (0.26 - 5.21) | 0.847 |
| Some difficulty | 0.62 (0.24 – 1.60) | 0.327 |
| Quite a lot of difficulty/ Cannot perform | 3.30 (0.52 – 20.99) | 0.205 |
|  |  |  |
| Picking up a kitchen chair and moving it, in order to clean |  |  |
| No difficulty | Reference |  |
| A little difficulty | 1.16 (0.47 - 2.85) | 0.753 |
| Some difficulty | 1.23 (0.15 – 10.37) | 0.847 |
| Quite a lot of difficulty/ Cannot perform | 1.27 (0.41 – 3.98) | 0.678 |
|  |  |  |
| Making a bed, including spreading and tucking in bed sheets |  |  |
| No difficulty | Reference |  |
| A little difficulty | 1.33 (0.53 – 3.31) | 0.540 |
| Some difficulty | 1.95 (0.58 – 6.57) | 0.279 |
| Quite a lot of difficulty/ Cannot perform | 1.32 (0.40 – 4.35) | 0.652 |
|  |  |  |
| Stepping on and off a bus |  |  |
| No difficulty | Reference |  |
| A little difficulty | 0.80 (0.12 – 5.26) | 0.819 |
| Some difficulty | 2.40 (0.98 – 5.86) | 0.055 |
| Quite a lot of difficulty/ Cannot perform | 3.24 (1.10, 9.52) | 0.033 |
|  |  |  |
| Washing dishes, pots and utensils by hand while standing at sink |  |  |
| No difficulty | Reference |  |
| A little difficulty | 0.88 (0.14 – 5.28) | 0.072 |
| Some difficulty | 0.63 (0.13 – 2.95) | 0.554 |
| Quite a lot of difficulty/ Cannot perform | 2.97 (0.91 – 9.72) | 0.072 |
|  |  |  |
| Walking around one floor of your home, taking into consideration doors, furniture |  |  |
| No difficulty | Reference |  |
| A little difficulty | 1.56 (0.46 – 5.28) | 0.471 |
| Some difficulty | 3.21 (0.61 – 16.92) | 0.169 |
| Quite a lot of difficulty/ Cannot perform | 1.84 (0.38, 8.80) | 0.445 |
|  |  |  |
|  | **IRR (95% CI)*** | **P-value** |
| **Advanced lower extremity functioning domain** |  |  |
|  |  |  |
| Walking several blocks |  |  |
| No difficulty | Reference |  |
| A little difficulty | 0.66 (0.18 – 2.42) | 0.535 |
| Some difficulty | 1.43 (0.33 – 6.27) | 0.965 |
| Quite a lot of difficulty/ Cannot perform | 1.50 (0.49 – 4.60) | 0.482 |
|  |  |  |
| Carrying something in both arms while climbing a flight of stairs |  |  |
| No difficulty | Reference |  |
| A little difficulty | 1.23 (0.53 – 2.84) | 0.624 |
| Some difficulty | 0.64 (0.16 – 2.57) | 0.531 |
| Quite a lot of difficulty/ Cannot perform | 2.00 (0.75 – 5.31) | 0.163 |
|  |  |  |
| Going up and down 3 flights of stairs using a handrail |  |  |
| No difficulty | Reference |  |
| A little difficulty | 1.94 (0.93 - 4.61) | 0.074 |
| Some difficulty | 0.30 (0.05 – 1.92) | 0.204 |
| Quite a lot of difficulty/ Cannot perform | 2.30 (0.80 – 6.63) | 0.122 |
|  |  |  |
| Hiking a couple of kilometres on uneven surfaces, including hills |  |  |
| No difficulty | Reference |  |
| A little difficulty | 1.68 (0.67 - 4.21) | 0.266 |
| Some difficulty | 1.65 (0.64 – 4.25) | 0.300 |
| Quite a lot of difficulty/ Cannot perform | 2.06 (0.83 – 5.11) | 0.120 |
|  |  |  |
| IRR: incidence rate ratio; CI: confidence interval; *Adjusted for age, gender, ethnic group, living arrangement, hypertension, polypharmacy, nutritional status, self-reported depression, stroke, osteoarthritis, osteoporosis, vision/hearing impairment, and dementia | | |
